# A successful conclusion to the long search for TRPV5 pathogenic variants in monogenic hypercalciuria

**DOI:** 10.1038/s41431-024-01613-y

**Published:** 2024-06-05

**Authors:** Caroline M. Gorvin

**Affiliations:** 1 Institute of Metabolism and Systems Research (IMSR) and Centre for Diabetes, Endocrinology and Metabolism (CEDAM), University of Birmingham, Birmingham, B15 2TT UK; 2 Centre for Membrane Proteins and Receptors (COMPARE), Universities of Birmingham and Nottingham, Birmingham, B15 2TT UK

**Keywords:** Endocrine system and metabolic diseases, Kidney diseases

Hypercalciuria, or excessive urinary calcium excretion, is the most common metabolic risk factor for kidney stone disease, occurring in up to 40% of stone formers, and can contribute to fragility fractures and osteoporosis. Hypercalciuria has an estimated heritability of ~50% and >25 genes have been linked to monogenic hypercalciuria, most of which occur as part of a syndrome with other biochemical abnormalities such as tubular proteinuria in Dent’s Disease, metabolic alkalosis in Bartter’s Syndrome or hypocalcaemia in autosomal dominant hypocalcaemia. Genome-wide association studies have also identified several susceptibility risk variants in genes involved in calcium transport [1, 2]. However, the molecular basis of most cases of hypercalciuria are unknown. In this issue, Guleray Lafci et al. demonstrate that a non-synonymous missense inactivating genetic variant in *TRPV5* (transient receptor potential cation channel subfamily V member 5) causes a novel form of autosomal recessive hypercalciuria [3], concluding a decades long search for *TRPV5* variants in heritable hypercalciuria.

*TRPV5*, located on chromosome 7q34, encodes a 729 amino acid calcium channel that forms a functional tetramer at cell surfaces. It is predominantly expressed at the apical membrane of the distal convoluted tubule and connecting tubule, where it has been described as the gatekeeper of active calcium reabsorption, regulating calcium’s entry to the cell. Following influx through TRPV5, calcium is transported to basolateral membranes via calbindin proteins, then exported by a sodium-calcium exchanger or Ca^2+^-ATPase. These processes, including TRPV5 expression and activity, undergo hormonal regulation by the parathyroid hormone (PTH) and 1,25(OH)_2_D_3_ [1]. Due to the vital role of TRPV5 in calcium reabsorption, associations between variants in the gene and renal hypercalciuria and kidney stone formation have been sought since the channel’s discovery twenty-five years ago. This was driven by findings in mice with global genetic ablation of Trpv5 that exhibit a severe hypercalciuria (6x that observed in wild-type mice) due to reduced renal calcium reabsorption [4], and a subsequent *N*-ethyl-*N*-nitrosourea (ENU)-induced mouse with autosomal dominant hypercalciuria due to a Trpv5 p.(Ser682Pro) variant that reduced baseline calcium permeability [2]. However, studies in human populations have, to date, been disappointing, with targeted sequencing of *TRPV5* in families with hypercalciuria identifying no significant findings [5, 6]. Genome-wide association studies of recurrent kidney stone formers also failed to identify a TRPV5 signal at the genome-wide level, but a rare missense variant (p.(Leu530Arg)) affecting the pore-forming region of TRPV5 was identified in a large case-control study from Iceland and significantly associated with risk of recurrent kidney stones [7]. Studies of individuals in Indian [8] and Taiwanese populations [9] have also reported associations between TRPV5 polymorphisms and urinary calcium excretion, familial stone disease or stone multiplicity. However, these polymorphisms have no effect on calcium channel activity [5] or have high allele frequencies (>0.2) in population databases such as GnomAD, indicating that they may not be causative of hypercalciuria in these individuals. Therefore, the identification of a biallelic inactivating TRPV5 variant p.(Val598Met) in multiple affected members of a family provides the first convincing evidence that TRPV5 contributes to monogenic hypercalciuria [3].

The variant identified p.(Val598Met) affects the TRP helix region of TRPV5, which controls channel pore gating, assembly and folding, and structural modelling predicted that the variant residue decreases protein flexibility. Calcium channel activity by electrophysiology was not performed. However, expression of the variant channel in HEK293 cells showed reduced calcium uptake, impaired glycosylation and reduced protein expression when compared to cells expressing wild-type TRPV5 [3]. Co-expression of cells with the variant protein and wild-type protein revealed that the p.(Val598Met) variant does not exert a dominant-negative effect on the wild-type channel [3].

The family with the variant had some unique characteristics that perhaps hint at why TRPV5 variants have remained elusive in previous studies. The three affected individuals were born to consanguineous parents and the variant was inherited as an autosomal recessive trait, with in vitro studies indicating that the variant does not elicit a dominant-negative effect [3]. Previous searches for TRPV5 variants have largely examined cases of hypercalciuria or kidney stone formers with a likely autosomal dominant inheritance. The current case and studies of the *Trpv5* knockout mouse and its heterozygous littermates indicate that a loss of functional channel may be required on both alleles for hypercalciuria with normal PTH to be present [4]. Thus, investigation of hypercalciuria or kidney stone disease in consanguineous families may yield more variants in the TRPV5 gene. However, there is some evidence that point mutations may contribute to hypercalciuria, as heterozygous mice with the ENU-induced TRPV5 p.(Ser682Pro) variant have hypercalciuria and hyperphosphaturia, similar to the family described with the p.(Val598Met) variant. Moreover, the p.(Leu530Arg) rare variant was identified in a targeted search of 2636 individuals from an Icelandic population with recurrent kidney stones, with no report of homozygous individuals. Functional analyses of the p.(Leu530Arg) variant showed impairments in calcium transport and glycosylation, similar to defects identified for the p.(Val598Met) variant identified in the monogenic hypercalciuria family [10], and provides further evidence that single-point mutations can have an impact on calcium channel activity. It is likely that studies of consanguineous families and very large cohorts by targeted sequencing rather than genome-wide searches may reveal more pathogenic variants in TRPV5.

The proband with the p.(Val598Met) TRPV5 variant also had additional symptoms that were not shared with his affected siblings or observed in mouse models. These included short stature, bone deformities, elevated alkaline phosphatase and PTH with normocalcaemia and normal vitamin D levels, which partially overlap, but are not wholly consistent with disorders including primary hyperparathyroidism and hereditary hypophosphataemic rickets. Further investigation of the proband revealed a somatic variant in *GNAS* (p.(Arg201Cys)), which is a well-characterised pathogenic variant known to cause fibrous dysplasia [3]. Therefore, it is likely that these additional bone phenotypes are due to the somatic *GNAS* variant, while the germline *TRPV5* variant causes hypercalciuria.

Studies of the family do have limitations. These include that the TRPV5 p.(Val598Met) variant is present on population databases such as GnomAD. However, all individuals with this variant are reported as heterozygous and thus in vitro studies would suggest should be normocalciuric. Additionally, the whole-exome sequencing did reveal additional genetic variants that were not functionally characterised. However, these either had limited expression in the kidney or previously reported knockout mouse studies had no phenotypic abnormalities. Moreover, while the variant was only found in a single hypercalciuric family, further studies of other consanguineous families with idiopathic hypercalciuria may provide more evidence that pathogenic TRPV5 variants contribute to human disease.
